# ELM—the Eukaryotic Linear Motif resource—2024 update

**DOI:** 10.1093/nar/gkad1058

**Published:** 2023-11-14

**Authors:** Manjeet Kumar, Sushama Michael, Jesús Alvarado-Valverde, András Zeke, Tamas Lazar, Juliana Glavina, Eszter Nagy-Kanta, Juan Mac Donagh, Zsofia E Kalman, Stefano Pascarelli, Nicolas Palopoli, László Dobson, Carmen Florencia Suarez, Kim Van Roey, Izabella Krystkowiak, Juan Esteban Griffin, Anurag Nagpal, Rajesh Bhardwaj, Francesca Diella, Bálint Mészáros, Kellie Dean, Norman E Davey, Rita Pancsa, Lucía B Chemes, Toby J Gibson

**Affiliations:** Structural and Computational Biology Unit, European Molecular Biology Laboratory, Heidelberg 69117, Germany; Structural and Computational Biology Unit, European Molecular Biology Laboratory, Heidelberg 69117, Germany; Structural and Computational Biology Unit, European Molecular Biology Laboratory, Heidelberg 69117, Germany; Collaboration for joint PhD degree between EMBL and Heidelberg University, Faculty of Biosciences, Germany; Institute of Enzymology, HUN-REN Research Centre for Natural Sciences, Budapest 1117, Hungary; VIB-VUB Center for Structural Biology, Vlaams Instituut voor Biotechnologie, Pleinlaan 2, 1050 Brussels, Belgium; Structural Biology Brussels, Department of Bioengineering, Vrije Universiteit Brussel, Pleinlaan 2, 1050 Brussels, Belgium; Instituto de Investigaciones Biotecnológicas, Universidad Nacional de San Martín (UNSAM), Consejo Nacional de Investigaciones Científicas y Técnicas (CONICET), CP 1650, Buenos Aires, Argentina; Escuela de Bio y Nanotecnologías (EByN), Universidad Nacional de San Martín, Av. 25 de Mayo y Francia, CP1650 San Martín, Buenos Aires, Argentina; Faculty of Information Technology and Bionics, Pázmány Péter Catholic University, Práter u. 50/A, Budapest 1083, Hungary; Departamento de Ciencia y Tecnología, Universidad Nacional de Quilmes - Consejo Nacional de Investigaciones Científicas y Técnicas (CONICET), Bernal, Buenos Aires, Argentina; Structural and Computational Biology Unit, European Molecular Biology Laboratory, Heidelberg 69117, Germany; Faculty of Information Technology and Bionics, Pázmány Péter Catholic University, Práter u. 50/A, Budapest 1083, Hungary; Department of Computational Biology, University of Lausanne, Lausanne, Switzerland; Department of Biology, Institute of Molecular Systems Biology, ETH Zurich, Zurich, Switzerland; Swiss Institute of Bioinformatics, Lausanne, Switzerland; Departamento de Ciencia y Tecnología, Universidad Nacional de Quilmes - Consejo Nacional de Investigaciones Científicas y Técnicas (CONICET), Bernal, Buenos Aires, Argentina; Structural and Computational Biology Unit, European Molecular Biology Laboratory, Heidelberg 69117, Germany; Department of Bioinformatics, Semmelweis University, Tűzoltó u. 7, Budapest 1094, Hungary; Instituto de Investigaciones Biotecnológicas, Universidad Nacional de San Martín (UNSAM), Consejo Nacional de Investigaciones Científicas y Técnicas (CONICET), CP 1650, Buenos Aires, Argentina; Escuela de Bio y Nanotecnologías (EByN), Universidad Nacional de San Martín, Av. 25 de Mayo y Francia, CP1650 San Martín, Buenos Aires, Argentina; Health Services Research, Sciensano, Brussels, Belgium; Institute of Cancer Research, Chester Beatty Laboratories, 237 Fulham Rd, Chelsea, London SW3 6JB, UK; Departamento de Ciencia y Tecnología, Universidad Nacional de Quilmes - Consejo Nacional de Investigaciones Científicas y Técnicas (CONICET), Bernal, Buenos Aires, Argentina; Department of Biological Sciences, BITS Pilani, K. K. Birla Goa campus, Zuarinagar, Goa 403726, India; Inselspital, University of Bern, Freiburgstrasse 15, CH-3010 Bern, Switzerland; Structural and Computational Biology Unit, European Molecular Biology Laboratory, Heidelberg 69117, Germany; Department of Structural Biology and Center of Excellence for Data Driven Discovery, St Jude Children's Research Hospital, 262 Danny Thomas Place, Memphis, TN 38105, USA; School of Biochemistry and Cell Biology, 3.91 Western Gateway Building, University College Cork, Cork, Ireland; Institute of Cancer Research, Chester Beatty Laboratories, 237 Fulham Rd, Chelsea, London SW3 6JB, UK; Institute of Enzymology, HUN-REN Research Centre for Natural Sciences, Budapest 1117, Hungary; Instituto de Investigaciones Biotecnológicas, Universidad Nacional de San Martín (UNSAM), Consejo Nacional de Investigaciones Científicas y Técnicas (CONICET), CP 1650, Buenos Aires, Argentina; Escuela de Bio y Nanotecnologías (EByN), Universidad Nacional de San Martín, Av. 25 de Mayo y Francia, CP1650 San Martín, Buenos Aires, Argentina; Structural and Computational Biology Unit, European Molecular Biology Laboratory, Heidelberg 69117, Germany

## Abstract

Short Linear Motifs (SLiMs) are the smallest structural and functional components of modular eukaryotic proteins. They are also the most abundant, especially when considering post-translational modifications. As well as being found throughout the cell as part of regulatory processes, SLiMs are extensively mimicked by intracellular pathogens. At the heart of the Eukaryotic Linear Motif (ELM) Resource is a representative (not comprehensive) database. The ELM entries are created by a growing community of skilled annotators and provide an introduction to linear motif functionality for biomedical researchers. The 2024 ELM update includes 346 novel motif instances in areas ranging from innate immunity to both protein and RNA degradation systems. In total, 39 classes of newly annotated motifs have been added, and another 17 existing entries have been updated in the database. The 2024 ELM release now includes 356 motif classes incorporating 4283 individual motif instances manually curated from 4274 scientific publications and including >700 links to experimentally determined 3D structures. In a recent development, the InterPro protein module resource now also includes ELM data. ELM is available at: http://elm.eu.org.

## Graphical Abstract

**Graphical Abstract:**
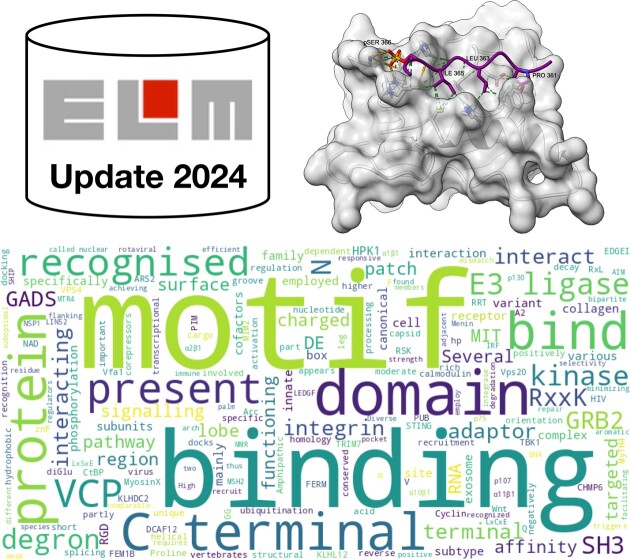

## Introduction

Many eukaryotic proteins are highly modular and have numerous structural and functional components. Multiple globular domains may be separated by substantial intrinsically disordered regions (IDRs) (1–4). Globular domains provide many functions, such as catalysis and high specificity macromolecular binding. IDR functions can be as simple as acting as linkers spacing out the folded domains. However, IDRs are also heavily involved in regulatory functions in all major signalling pathways, while often being modified by phosphorylation and other post-translational modifications (PTMs) acting as signals/switches, for example, of cell state (5,6). The smallest functional modules in eukaryotic proteins are the short linear motifs (SLiMs), typically 3–15 amino acids in length and being present primarily within IDRs and thus simultaneously providing flexibility and accessibility for interactions (7–9). When interacting with their partner protein domains, SLiMs are likely to acquire transiently stable conformations by the mechanisms of conformational selection or induced fit to their partner's interaction surface (10,11). Short linear motifs are considered to be by far the most abundant class of protein modules (12), with the corollary that the functions of most SLiM instances are yet to be elucidated.

SLiMs were first defined in 1990 by Tim Hunt (13), based on a few examples of subcellular localisation targeting signals, such as the KDEL endoplasmic reticulum (ER) retention motif (14), the positively charged nuclear targeting sequence (15,16) and peroxisomal targeting sequences (17). Over time, it has become clear that essentially all aspects of cell biology involve SLiM interactions (18). For example, they have key roles in cell cycle, vesicle trafficking, cytoskeleton dynamics, innate immunity and protein and RNA degradation systems (19–21). SLiMs bind partner protein domains with moderate affinity and therefore in many contexts they exhibit cooperativity to achieve their functional output (22–24). In this regard, multivalent SLiM-domain interactions appear to be important in liquid-liquid phase separation (LLPS) processes (25,26).

Many PTMs on SLiM targets are catalysed by amino acid residue-modifying enzymes such as protein kinases. For heavily researched kinases, the sequence specificities have been determined at the substrate sites, often aided by the use of SPOT array phosphorylation assays (27). However, for most of the > 530 human kinases, there has been little or no attempt to establish their phosphorylation site motifs. As a result, annotation of kinase specificities in ELM has been hampered. Nonetheless, a recent systematic attempt to provide SPOT arrays for the serine/threonine human kinome has been carried out by Johnson *et al.* (28). This will allow this important class of SLiMs to be better defined in future ELM updates.

The abundant use of SLiMs in cell regulation has a downside because they can be mimicked by intracellular pathogens, enabling the hijacking of cellular systems and thereby repurposing the host cell for pathogen reproduction. Many SLiMs such as the nuclear targeting sequence and the LxCxE-binding motif were first identified in viral proteins (16,29), with cellular counterparts only being discovered later (30,31).

Much of the experimental research on SLiM functions is focused on single protein instances. Structure determination and various types of *in vitro* peptide interaction/modification assays are helpful in establishing the amino acid preferences of the motifs. However, these approaches do not scale well. Therefore, there is growing interest in the deployment of more systematic approaches such as Phage Display (32), Holdup Assays (33) and large-scale and quantitative screening of linear motif binding specificity (34,35), recently reviewed by Davey *et al.* (36). These methods enable the discovery of large numbers of candidate SLiMs in the intrinsically disordered proteomes of hosts and pathogens that can be followed up with functional validation methods.

In this paper, we summarise the current status of the ELM resource in 2024 and highlight areas of data growth since the last report in 2022 (37).

## The ELM resource

The ELM resource provides an intensive platform to catalogue and explore the intricacies of SLiMs in proteins. The motif knowledge in the ELM database (ELM DB) is classified into six broad categories: cleavage (CLV), degradation (DEG), docking (DOC), ligand (LIG), modification (MOD) and targeting (TRG). Within themselves, each of these categories comprises high-level functionally similar motif classes/entries.

The core of each motif entry is built from a detailed compilation of motif instances, which are rigorously extracted from existing scientific studies. In the ELM DB, these instances hold fine-grained details of annotated evidence, including methods used to characterise the motif as well as the curator's inference on the reliability of annotated data (38). The instances are also analysed from various aspects, such as sequence, structure, function, localisation, evolution and interaction context, to define the primary amino acid residues contributing to the specificity and binding strength of the motif-mediated protein complex. This systematic analysis by the ELM curators enables defining the motif sequence pattern in a standard POSIX regular expression format (detailed in Wikipedia (https://en.wikipedia.org/wiki/Regular_expression) and the ELM website for an in-depth view of their consensus definitions). A motif class also includes local sequence context and molecular interaction features from the core motif positions. Moreover, each class also assimilates critical insights into the biological role as well as the contextual basis for supporting the presence of a motif in the given protein sequence. Overall, every class distributes several key biological insights for the motif such as its functional attributes, cellular context, interacting domain knowledge, cellular locations, and curated knowledge from the scientific literature.

ELM curations are systematically organised in the PostgreSQL relational database (http://www.postgresql.org/) backend and can be updated as new knowledge or findings become available for any existing motif entry. The ELM DB can be queried through the Django web framework (https://djangoproject.com/), and output is presented via its front-end interface. The ELM resource also provides access to a toolkit for motif exploration, allowing users to identify potential motifs in proteins of their interest. Once the query sequence matches with the known motif patterns in the ELM DB, they are displayed graphically on the protein sequence. The motif matches are overlaid with contextual features, which include accessibility and conservation information, among other functional insights.

The curated knowledge within the ELM repository can be accessed and downloaded for free in various formats. Detailed specifications on the available formats and datasets are available at http://elm.eu.org/downloads.html. Furthermore, a REST-API is available to facilitate automated queries of ELM class consensus matches against proteins of interest. The access details for utilising the motif search REST-API can be found at http://elm.eu.org/api/manual.html.

## Data updates in ELM

**Figure 1.**
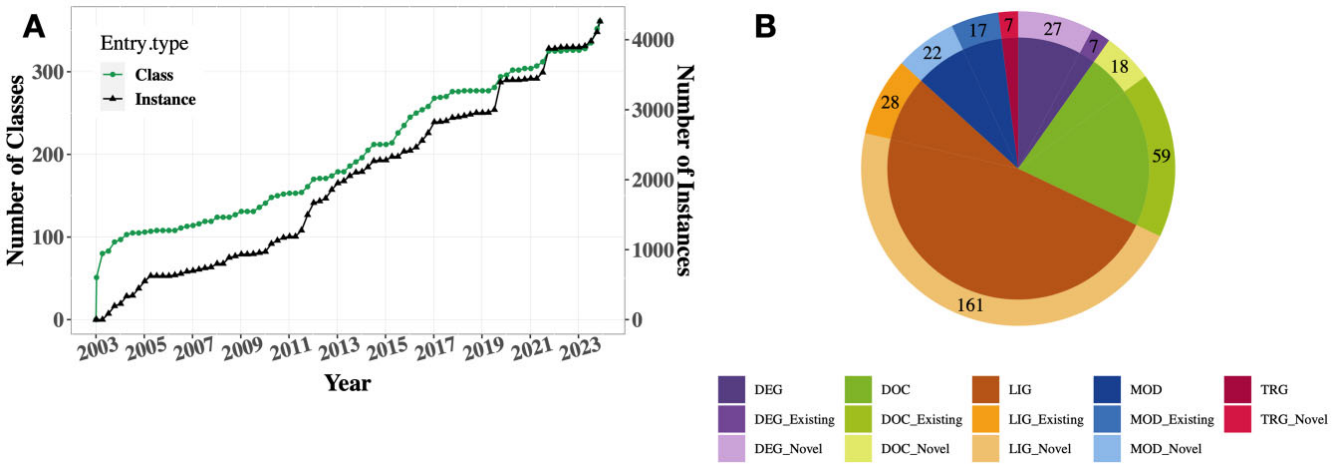
Data growth trends within the ELM resource since the last update paper (37). (**A**) This depicts the cumulative count of both motif classes and instances inserted into ELM in the last two decades. (**B**) The pie chart illustrates the allocation of new instances among both new and updated ELM classes (lighter and darker shades in the outer circle, respectively) belonging to 5 different functional categories. Note that the pie chart focuses only on the classes where new instances were added in this update. Visualisations were done using ggplot2 in RStudio (http://www.rstudio.com/).

Since its 2022 update (37), ELM curation has primarily focused on motifs pivotal to RNA and protein degradation, innate immunity, kinase biology, synapse systems, the cell cycle and pathogen-host interactions, among other motif-governed areas. With this update, ELM catalogues 356 motif classes (Figure 1; Table 1)—an addition of 39 since the last release (Table 2)—with a total of 4283 annotated motif instances, resulting in a net increase of 346 instances. Additionally, 17 existing motif classes and 111 instances have been updated based on recent research findings (Table 2). Major advances in the updated motif classes were focused on improvements of the regular expressions defining the motif specificity and on the inclusion of structural and affinity data for each motif. ELM now maintains links to 4274 scientific publications. The growth in protein structure information in ELM has reached a new high and with this update 102 structures have been integrated, bringing the total cross-referenced structures with databases such as PDBe and RCSB-PDB to 718. Additionally, the ELM dataset now defines 2749 motif-partner interactions, of which 688 have curated binding affinities. In-depth insights into ELM’s data are provided in Table 1.

**Table 1.** Concise summary of data captured in the ELM resource

Functional sites		ELM classes		ELM instances		GO terms		PDB structures	PubMed links
**Total**	**220**	**356**		**4283**		**1018**		**718**	**4274**
By Category		LIG	201	Human	2459	Biological Process	567		
		MOD	40	Mouse	404				
		DOC	43	Rat	174	Cellular Component	220		
		DEG	33	Yeast	380				
		TRG	28	Viruses	290	Molecular Function	231		
		CLV	11	Others	576				

**Table 2.** New and revised ELM entries in the current ELM release

ELM class identifier	#Instances	ELM class (short) description
**New ELM Classes**		
DEG_Cend_DCAF12_1	6	C-terminal diGlu degrons recognised by the DCAF12 E3 ligase
DEG_Cend_FEM1AC_1	-	C-terminal degrons recognised by the FEM1A and FEM1C E3 ligases
DEG_Cend_FEM1B_2	2	C-terminal degrons recognised by the FEM1B E3 ligase
DEG_Cend_KLHDC2_1	2	C-terminal GG degrons targeted by the KLHDC2 E3 ligase
DEG_Cend_TRIM7_1	6	C-terminal Q degrons targeted by the TRIM7 E3 ligase
DEG_CRBN_cyclicCter_1	-	Degron recognised by the thalidomide-binding domain (TBD) of cereblon for ubiquitination and subsequent proteasomal degradation
DEG_Kelch_KLHL12_1	11	Proline-rich degron of the Wnt signalling pathway recognised by the E3 ligase KLHL12 with moderate affinity
DOC_CYCLIN_RevRxL_6	1	The hydrophobic patch (hp) of Cyclin A2 can bind an RxL-like motif in the reverse orientation
DOC_MIT_MIM_2	5	MIT- interacting motif (MIM2) present in CHMP6, Vfa1, Vps20 docks at MIT domain present in VPS4
DOC_PUB_PIM_1	2	The PIM motif binds to the PUB domain of proteins involved in the regulation of ubiquitination
DOC_RSK_DDVF_1	5	This short, partly acid [DE]-[DE]-V-F motif binds to the surface region of the RSK N-terminal kinase domain
DOC_TBK1_STING_1	5	A TBK1 kinase recruitment and activation docking motif conserved in STING
LIG_Arc_Nlobe_1	11	Binding motif for the N-lobe part of the C-terminal domain in Arc. The N-lobe domain has structural homology with HIV virus capsid, but the binding appears to be unique to higher vertebrates
LIG_ARS2_EDGEI_1	21	Several proteins functioning in RNA decay/processing/splicing bind a positively charged patch on the C-terminal leg domain (a znF) of ARS2 through their negatively charged EDGEI motif
LIG_CaM_Basic-1–5-8–14_1	4	Amphipathic α-helical calmodulin binding motif
LIG_CtBP_RRT_2	5	The RRT motif binds to a groove on the nucleotide binding domain of CtBP proteins functioning as NAD-dependent transcriptional corepressors
LIG_FERM_MyoX_1	4	Motif specifically recognised by the MyTH4-FERM domain of MyosinX that is important for cargo recognition
LIG_Integrin_collagen_1	7	A collagen-specific binding motif that is recognized by the I-domain of collagen binding integrins α1β1, α2β1, α10β1, α11β1
LIG_Integrin_RGDSP_6	6	A variant of the canonical RGD motif minimizing flanking interactions with the integrin, thus achieving weak selectivity and comparable binding strength over all RGD-binding integrins
LIG_IRF7_LxLS_2	1	A binding site for IRF-7 present in the protein itself, in various innate adaptor proteins, and in rotaviral NSP1 which triggers the innate immune responsive pathways
LIG_LEDGF_IBM_1	9	A bipartite motif recognised by the integrase binding domain of LEDGF/p75
LIG_Menin_MBM1_1	4	High affinity motif recognised by the palm region of Menin protein
LIG_MSH2_SHIPbox_1	10	The aromatic SHIP box motif is employed by several positive regulators of DNA mismatch repair (MMR) to interact with MSH2
LIG_MTR4_AIM_1	8	Diverse nuclear exosome adaptors employ the so-called arch-interacting motif (AIM) to recruit the MTR4-exosome complex to different RNA species for facilitating their efficient degradation
LIG_RBL1_LxSxE_2	1	The LxSxE motif is a suboptimal variant of the LxCxE motif found in the LIN52 protein that interacts with two members of the pocket protein family (p107 and p130). Binding requires phosphorylation of an adjacent residue creating a phosphoswitch
LIG_SH2_SFK_CTail_3	4	Internal SH2 binding motif in SRC family kinase C-terminal tails
LIG_SH3_PxRPPK_7	6	This PxRPxK subtype of the RxxK motifs is typically employed by GRB2-associated-binding proteins (GABs) 1, 2 and 3, and by other proteins, such as THEMIS, for binding of the C-terminal SH3 domains of GRB2 or GADS
LIG_SH3_PxxPPRxxK_8	6	This HPK1 subtype of the RxxK motifs, where the RxxK is preceded by an upstream PxxP, is used by HPK1 and some other proteins mainly in T-cell receptor signalling to interact with the C-terminal SH3 domain of GADS or GRB2
LIG_SH3_PxxxRxxKP_6	16	The C-terminal SH3 domains of GADS and GRB2, and the SH3s of STAM1 and STAM2 have been described to bind this canonical RxxK motif
LIG_TRAF3_MATH_PxP_3	2	A motif that specifically binds the TRAF3 E3 ligase
LIG_TRAF4_MATH_1	3	A TRAF4 MATH domain binding motif present in some platelet receptors
LIG_Trf4_IWRxY_1	4	TRAMP complex subunits Air1/2 bind subunits Trf4/Trf5 through an extended interaction surface, involving the IWRxY motif
LIG_VCP_SHPBox_1	17	The SHP box motif is a VCP-binding ligand present in some adaptors that bind to the C-terminal NTD subdomain of VCP
LIG_VCP_VBM_3	3	The VCP Binding Motif (VBM) binds to the N-terminal domain of VCP and is present in some of its cofactors.
LIG_VCP_VIM_2	9	VCP Interacting Motif (VIM) binds to the N-terminal domain of VCP and is present in various cofactors
MOD_AAK1BIKe_LxxQxTG_1	17	Motif that is phosphorylated by the endocytic kinases AAK1 and/or BIKe
MOD_LOK_YxT_1	5	The optimal phosphorylation site of the basophilic lymphocyte-oriented kinase (LOK) and SLK that mainly regulate cell shape and motility
TRG_NESrev_CRM1_2	2	Reverse NES binding the CRM1 groove in the minus direction. The spacing of the initial two hydrophobic residues ΦxΦ dictates the reverse orientation
TRG_Oom_RxLR_1	5	Oomycete host targeting signal
**Extensively Revised ELM Classes**		
DEG_APCC_DBOX_1	18	An RxxL-based motif that binds to the Cdh1 and Cdc20 components of APC/C thereby targeting the protein for destruction in a cell cycle dependent manner
DOC_PP2B_LxvP_1	50	Docking motif in calcineurin substrates that binds at the interface of the catalytic CNA and regulatory CNB subunits.
DOC_PP2B_PxIxIT_1	27	PxIxIT docking motif in calcineurin substrates that binds the catalytic CNA subunit
LIG_CaMK_CASK_1	7	Motif that mediates binding to the calmodulin-dependent protein kinase (CaMK) domain of the peripheral plasma membrane protein CASK/Lin2
LIG_IRFs_LxIS_1	9	A binding site for proteins IRF-3, IRF-5 and IRF-6 present in the protein themselves, in various innate adaptor proteins, and in rotaviral NSP1 which triggers the innate immune responsive pathways
LIG_Nrd1CID_NIM_1	5	The CTD-interacting domain (CID) of Nrd1 interacts with the NIM motif in Trf4 and some other proteins in ncRNA degradation
LIG_RB_LxCxE_1	45	The LxCxE motif is found in multiple host and viral interactors of the pocket protein family (Rb, p107 and p130)
LIG_RB_pABgroove_1	7	The LxDLFD motif binds in a deep groove between the A and B subdomains of the Retinoblastoma (Rb), p107 and p130 pocket domains
LIG_SH2_NCK_1	17	NCK Src Homology 2 (SH2) domain binding motif
LIG_SH2_SFK_2	20	Phosphotyrosine motifs bound by SH2 domains of Src family kinases (SFKs)
LIG_SH3_PxxDY_5	7	The PxxDY motif is recognized by some SH3 domains including in Nck and Eps8
LIG_TRAF2like_MATH_loPxQ_2	5	Long (or minor) TRAF2 binding motif. Members of the tumour necrosis factor receptor (TNFR) superfamily initiate intracellular signaling by recruiting the C-domain of the TNFR-associated factors (TRAFs) to their cytoplasmic tails
LIG_TRAF2like_MATH_shPxQ_1	13	Short (or major) TRAF2 binding motif. Members of the tumour necrosis factor receptor (TNFR) superfamily initiate intracellular signaling by recruiting the C-domain of the TNFR-associated factors (TRAFs) to their cytosolic tails
LIG_TRAF6_MATH_1	34	TRAF6 binding site. Members of the tumour necrosis factor receptor (TNFR) superfamily initiate intracellular signalling by recruiting the C-domains of the TNFR-associated factors (TRAFs) using motifs in their cytoplasmic tails
MOD_NEK2_1	2	Strict version of the motif targeted by NEK2 for phosphorylation
MOD_NEK2_2	-	The more tolerant version of the Nek2 phosphosite motif
MOD_PIKK_1	48	(ST)Q motif which is phosphorylated by PIKK family members

## A selection of newly added motif biology insights covered in the current ELM update

### SLiMs in RNA processing and decay

In eukaryotic cells, numerous RNA processing and modification events are required to produce RNAs in their final functional forms. To remove defective, unstable or unwanted transcripts from the nucleus, RNAs must first be identified and then degraded by the nuclear exosome (39), a multi-component protein complex of RNA processing enzymes.

**Figure 2.**
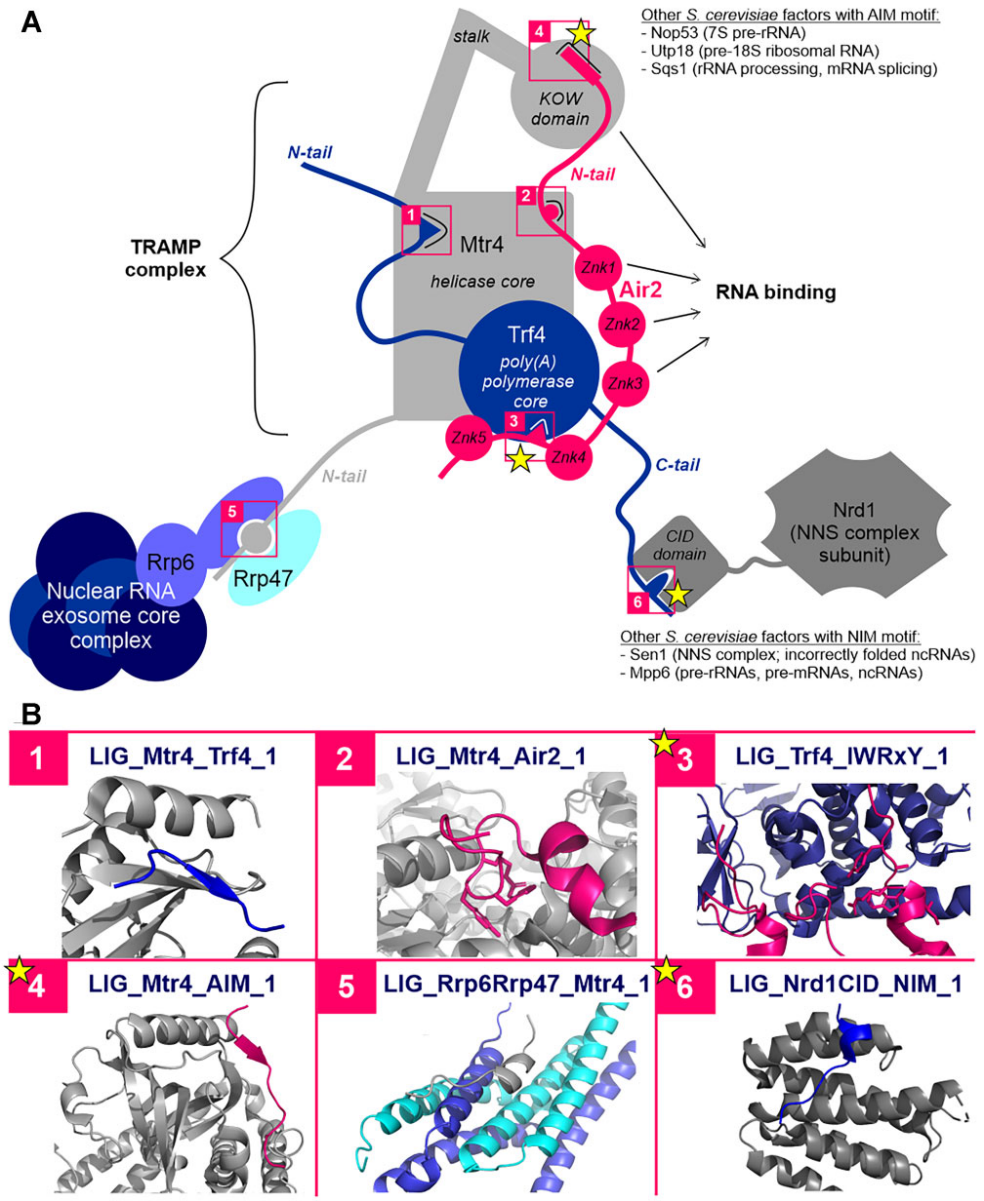
SLiMs in nuclear RNA processing and decay. (**A**) Schematic representation of the motif-mediated interactions maintaining the inter-subunit interactions of the TRAMP complex and facilitating the recruitment of the complex and the associated exosome to different types of degradable RNA. Motif-mediated interactions are highlighted with numbered pink boxes, with the ones newly added or extensively revised since the last release marked by a star. Where the given motif is employed by several yeast factors belonging to different protein families, the factors and their respective bound RNA types are listed. Abbreviations: CID, CTD-interacting domain, Znk, zinc knuckle, ncRNA - non-coding RNA, AIM, arch-interacting motif, NIM, Nrd1-interacting motif. (**B**) Structural snapshots of the numbered motif-mediated interactions. Colours for the domains and/or motifs depicted in each box match those of the corresponding proteins in the schematic figure of panel A. Within each box, all protein domains are depicted in cartoon representation. In most cases (boxes 1, 4, 5 and 6) cartoon representation was also applied to the SLiMs for simplicity. In the case of more extensive binding interfaces (boxes 2 and 3), where the motif residues would be difficult to guess, residues belonging to the SLiMs are shown in stick representation. The structural snapshots do not always show the interaction of the exact same proteins as depicted in panel A, in some cases, an X-ray structure was only available for the same type of motif from another protein. (1) PDB: 4U4C (Mtr4 with a Trf4-Air2 fusion protein, Trf4 motif depicted); (2) PDB:4U4C (Mtr4 with a Trf4-Air2 fusion protein, Air2 motif depicted); (3) PDB:3NYB (Trf4 with Air2 regions including the 4th and 5th zink knuckles and the motif in the linker connecting those; motif residues in stick representation); (4) PDB:5OOQ (Mtr4 KOW domain with the AIM motif from Nop53); (5) PDB:4WFD (the PMC2NT domain of Rrp6 forms a heterodimer with cofactor Rrp47 and the resulting dimer interface binds the motif within the N-terminal tail of Mtr4); (6) PDB: 6O3W (CID domain of Nrd1 bound by a Sen1 NIM motif).

The RNA helicase Mtr4 is an important cofactor of the nuclear exosome (20,40,41). Mtr4 is not just a helicase but rather a multi-domain, modular hub protein (20) that contributes to the processing of ribosomal RNAs (42) and the targeting of the exosome to different degradable RNA species (40). Mtr4 is part of the TRAMP complex, together with the Trf4 poly(A)polymerase and the Air2 RNA-binding adaptor protein (40,43). Most of the hitherto discovered SLiMs associated with nuclear RNA surveillance are either mediating the inter-subunit interactions of the TRAMP complex or facilitating the recruitment of the complex by diverse nuclear exosome cofactors (Figure 2).

A highly conserved motif within the N-terminal tail of yeast Trf4 interacts with the helicase core of Mtr4 (see the LIG_Mtr4_Trf4_1 motif class; (40)). This interaction is conserved in vertebrates, although with slightly different motif sequence preferences (see LIG_Mtr4_Trf4_2). Within the N-terminal tail of Air2 there are two distinct Mtr4-interacting motifs, one interacts with the helicase core (LIG_Mtr4_Air2_1) (40), while the other can bind to the KOW domain within the arch of Mtr4 (LIG_Mtr4_AIM_1) (40). This newly added, so-called arch-interacting motif (AIM; LIG_Mtr4_AIM_1) is particularly important, because many nuclear exosome cofactors, such as Nop53, Utp18 and Sqs1 also employ this motif for binding to and recruiting Mtr4 and the exosome to specific RNA transcripts to be degraded (41,44). Furthermore, this mode of interaction is also conserved in vertebrates (20,45). In the TRAMP complex, the Air proteins, Air1 or Air2, interact with Trf4 through a conserved motif within the linker between their fourth and fifth zinc knuckles (LIG_Trf4_IWRxY_1) plus their fifth zinc knuckle itself (46,47). Trf4 also contributes to the targeting of complexes for the degradation of incorrectly folded non-coding RNAs. The C-terminal Nrd1-interacting motif (NIM) of Trf4 binds to the NNS (Nrd1-Nab3-Sen1) complex subunit Nrd1 (48). This NIM motif class (LIG_Nrd1CID_NIM_1) has now been extensively revised, since the NNS subunit Sen1 (49) and exosome-associated RNA-binding protein Mpp6 (50) also turned out to employ NIM motifs for the binding of Nrd1.

Besides the TRAMP complex, additional RNA processing complexes have been characterised. In the yeast *S. pombe*, the MTREC (Mtl1–Red1 core) complex is responsible for directing the degradation of meiotic, non-coding and unspliced RNAs (51–53). Mtl1 (Mtr4-like protein 1) is a homolog of Mtr4. Red1 protein (54) interacts with Mtr1 and additional proteins in the MTREC complex, including zinc-finger containing protein Ars2 (52). Red1 contains an EDGEI motif (see newly annotated class LIG_ARS2_EDGEI_1) that is responsible for its interaction with Ars2.

In humans, there are a myriad of binding partners that interact with the human homolog of yeast Ars2, the Serrate RNA effector molecule homolog protein, through one or more EDGEI motifs. The binding of these partners facilitates different RNA processing activities (55–57). Interestingly, there are examples of EDGEI-mediated interactions even in *A. thaliana* and *D. melanogaster*; therefore they seem to be highly conserved across evolution.

### Protein degradation

**Figure 3.**
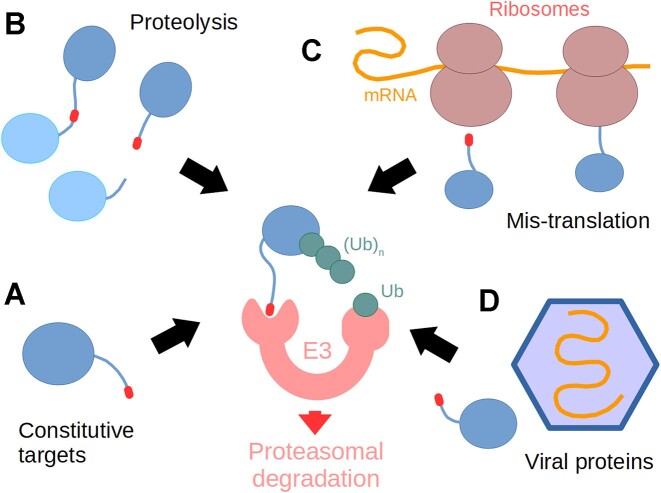
The diverse functions of C-terminal degrons. These motifs (red) can be part of native, endogenous protein C-termini (**A**), or be at an internal location and exposed by proteolysis (**B**). Degron SLiMs may also originate from erroneously translated mRNA sequences (**C**) or be present in virus-encoded proteins (**D**). These degrons are subsequently recognized by specific ubiquitin ligases (E3) and the proteins are polyubiquitylated, targeting them into the proteasome.

Protein quality control mechanisms are of quintessential importance to all living cells and their representation is becoming increasingly important for the ELM resource. Targeted degradation of proteins can remove unwanted, mis-translated polypeptides, improperly localised, fragmented or misfolded proteins, proteins from viral intruders, and many more (Figure 3) (58). Protein degradation within the eukaryotic cytoplasm and nucleus (and even of proteins translocated from the endoplasmic reticulum) is performed by the ubiquitin-proteasome system (59). Ubiquitination is orchestrated by at least three families of enzymes, E1 ubiquitin activating and E2 and E3 ubiquitin conjugating enzymes, which act sequentially, in distinct complexes (60) to add lysine 48 (K48)-linked polyubiquitin chains to the target protein. E3 ligases recognise their target proteins through molecular features called degrons. The majority of known degrons are SLiMs, and many of them are located at the protein amino (N-degrons) or carboxy (C-degrons) terminus (61). Most N‐terminal degrons are well known (explaining the so-called ‘N-end-rule’ determining protein half-life in eukaryotic cells) (62). However, C-degron pathways that selectively degrade proteins depending on the amino acids preceding their C-termini, also referred to as DesCEND (Destruction via C‐End Degron) pathways, have also been identified in recent years (58). The existence of C-terminal degrons explains why the distribution of C-terminal amino acids is unevenly biased in the proteome, as protein evolution actively selects against destabilising residues unless functionally required (63). So far, terminal Gly, Ala, Arg and Glu residues (‘destabilising residues’) are known to form parts of C-end degrons, being recognised by specific ubiquitin ligases (62,63).

C‐degron SLiMs consist of a few (typically only 2) conserved residues. A C‐terminal location and high structural accessibility (local disorder) are essential for C‐degrons to trigger degradation (58). C-terminal degrons can sometimes be present at the native C-termini of proteins or can be introduced by alternative mechanisms including premature translation termination or post-translational modifications (64,65). Terminal degron signals can also initially be internal, being latently present within the protein sequence and becoming exposed following proteolytic cleavage. E3 ligases recognising C-end degrons can be single- or multi-subunit molecules, like cullin-RING ligases (CRLs). The list of ubiquitin ligases and recognition subunits known to be associated with DesCEND pathways is extensive: KLHDC1/2/3/10, FEM1A/B/C, DCAF12, APPBP2, TRPC4AP, TRIM7, PIRH2, etc. (63,64,66,67). Unfortunately, not all E3 ligase substrate preferences have been sufficiently explored. The current ELM update includes new motif class definitions for the C-end recognition motifs of the DCAF12, FEM1A, FEM1B, FEM1C, KLHDC2 and TRIM7 ubiquitin E3 ligases (Table 2). Below we briefly discuss the DCAF12 and KLHDC2 cases.

The cullin 4-RING (CRL4) ubiquitin ligases are highly modular and associate to various substrate recognition subunits (termed DCAFs) through their shared subunit DDB1. DCAF12 has been found to recruit proteins with a C-terminal di-Glu motif (Glu-Glu-COOH). Known substrates of CRL4^DCAF12^ Cullin-RING E3 ligase include CCT5, a member of the T-complex protein ring (TRiC) chaperonin complex (68), as well as the MAGE-A3 and MAGE-A6 melanoma-associated antigens, involved in the regulation of ubiquitylation and autophagy (69). In all these examples, the extreme C-terminus of the substrate protein is bound tightly at the center of the WD40 domain of DCAF12, by a positively charged coronet of lysine (K91, K108, K254 in PDB: 8AJM/8AJO) and arginine (R203, R256, R344 in PDB: 8AJM/8AJO) residues (68). However, other, yet-unknown recognition modes might also exist for DCAF12, explaining its interaction with IAPs helping cell survival (70) or MOV10 involved in multiple cellular roles (71).

The KLHDC1, KLHDC2, KLHDC3 and KLHDC10 proteins are closely related vertebrate cullin 2 (Cul2) or cullin 5 (Cul5) RING (CRL2/CRL5) ubiquitin ligase substrate recognition subunits, binding C-terminal degrons ending in glycine with distinct but overlapping specificities. Out of this group, KLHDC2 is the best characterised protein, associated with Cul2, responsible for the recognition of substrates ending in a di-Gly (Gly-Gly-COOH) motif. Structural studies reveal that the degron is bound to the center of the Kelch repeat domain, with the twin arginines (R236 and R241 in PDB: 6DO3/6DO4/6DO5) coordinating the C-terminal carboxylate (65). The size of this pocket is restricted to binding either GG$ or (rarely seen) GA$ motifs (72). The last 6 amino acids also contact the surface, but there is relatively less restriction imposed on them, except for a preference for small side chains.

Interestingly, Di-Gly degrons (or Gly-Ala degrons), substrates of CRL2^KLHDC2^ can be generated by at least three distinct physiological processes. Some rare proteins natively end in two glycines, such as EPHB2 (73). These motifs can also be generated by proteolysis, as they are compatible with the substrates processed by certain ubiquitin-specific proteases, including an autoprocessing site in USP1 (65). Several selenoproteins can also suffer premature termination in case of selenium starvation, precluding the physiological overriding of the stop codons by selenocysteinyl-tRNAs (74). Selenoprotein K (SelK) and Selenoprotein S (SelS), when terminated prematurely, will end in a di-Gly motif, that is targeted by KLHDC2 (as well as other, related subunits) for ubiquitylation and destruction (65,74). Finally, KLHDC2 has also been shown to recognize its C-terminus (Gly-Ser-COOH) due to its resemblance to a degron, leading to the formation of inactive oligomers (73), adding a further layer of regulation to this already complicated system.

### Endoplasmic reticulum (ER)-associated degradation (ERAD)

Similar to degron-mediated protein destruction, the endoplasmic reticulum (ER)-associated degradation (ERAD) pathway functions as a guardian of exported and vesicular protein quality. This pathway differentiates between folded and misfolded proteins inside the ER lumen (75) and shuttles problematic proteins into the cytosol through membrane channels in an energy-dependent manner. Therefore, malfunctioning of the ERAD pathway can lead to the accumulation of defective proteins, a condition implicated in >60 diseases that include neurological disorders, cancer, and cystic fibrosis (76). Moreover, many viruses, including several Nidovirales species such as coronaviruses, hijack the ERAD machinery during viral replication while simultaneously evading immune detection by the host. Viruses exploit ER membrane-derived structures, such as double-membrane vesicles (DMVs), to conceal viral RNA from cytosolic cellular sensors responsible for initiating interferon production and innate immunity responses (77,78).

Prior to their elimination, most ERAD substrates are tagged with ubiquitin chains, which then serve as docking sites for VCP (Valosin-Containing Protein, also known as P97/TERA). VCP is an abundant protein (among the top 5% of cellular proteins) of the AAA+-ATPase group. It regulates various cellular processes, ranging from protein quality control to supporting DNA damage response and membrane trafficking. Importantly, VCP mutations have been linked to diseases characterised by protein aggregation, including Multisystem Proteinopathy (MSP), Familial Amyotrophic Lateral Sclerosis (fALS), and Charcot-Marie-Tooth Disease Type2Y (CMT2Y) (79). Essential to VCP’s interactions is its N-terminal domain (VCPN), which acts as a hub for binding various adaptor proteins. These interactions happen through three known motifs: VIM (VCP-Interacting Motif), VBM (VCP-Binding Motif), and the SHP box. These three motifs have been added as new ELM classes in the current update (Table 2). Notably, the VIM motif is hallmarked by a sequence module flanked on both sides with arginine preferences and a hydrophobic centre with conserved alanine residues. The VBM motif shows similarities with VIM in its arginine-rich amino acid content and interacts with the exact same VCP surface. In contrast, the SHP box forms a short, antiparallel β-strand, which augments the β-sheet of VCP’s NTD subdomain at a site distinct from the two other VCP-binding motifs. VCP itself has the newly annotated Pub domain Interacting (PIM) motif that docks to the E3 ligase HOIL-1-interacting protein (HOIP), a part of the Linear ubiquitin chain assembly complex (LUBAC) which modulates the NF-κB pathway (80).

### Update to the Kinase substrate specificities in ELM

In the current ELM update, we have added two new classes that define the phosphorylation site specificity of two kinases. One class represents the substrates of two Ser/Thr directed kinases, Adaptor-Associated kinase 1 (AAK1) and BMP-2 Inducible Kinase (BIKe/BMP2K). The second class defines the specificity of the Lymphocyte-oriented kinase (LOK), also named STK10 (Table 2).

AAK1 and BIKe/BMP2K are endocytic kinases that regulate diverse cellular functions, including receptor-mediated endocytosis, Notch pathway regulation, and dendrite morphogenesis. These kinases preferentially phosphorylate threonine residues and phosphorylate the QxTG motif present in the adaptor-related protein complex mu2 subunit (AP2M1) (81). Endocytic functions of these kinases also enable them to play a crucial role in controlling intracellular trafficking during the SARS-CoV-2 infection and entry process (82). Hepatitis C virus (HCV) and dengue virus (DENV) are other examples where these kinases are involved in viral entry and assembly/egress processes (83,84). The motif preference of the AAK1 and BIKe kinases is known from peptide SPOT arrays and other low-throughput in-vitro kinase assays (28,81,84–87) and indicates a strong preference for a Gly residue at the T + 1 position, Gln at the T – 2 position and Ile at the T – 5 position. Prk1p, the yeast homolog of AAK1/BIKe kinases, phosphorylates the Pan1p, Sla1p, and Scd5p substrates which all contain a similar phosphorylation motif (88–90).

LOK/STK10 regulates cytoskeletal dynamics in addition to affecting the cell shape and movements. An example of the latter includes its role in lymphocyte migration and microvilli formation in epithelial cells, which is achieved via phosphorylating the ERM proteins (Ezrin, Radixin and Moesin). Defining the specificity determinants of the LOK phosphosites is challenging due to the high sequence conservation of the C-terminal ERM protein regions, which contain the target phosphosites. Nevertheless, experiments from Belkina *et al.* (91) and SPOT arrays from Johnson JL *et al.* (28) reveal that LOK preferentially phosphorylates Thr over Ser residues. LOK sequence preferences around the phosphosite include Tyr at the T-2 position, large hydrophobic or aromatic amino acids at the T + 1 position and positively charged amino acids in the T – 3 and T + 2 positions flanking the phosphosite.

In addition to the two kinase classes with Thr site-specificity, a substantial update has been done to three classes describing the specificity for NEK (MOD_NEK2_1 and MOD_NEK2_2) and DNA damage signalling (MOD_PIKK_1) kinases. (Table 2). The ELM kinome update was assisted by the availability of a recent comprehensive assessment of the human Ser/Thr kinome using SPOT array technology (28), where the phosphosite preferences for 303 kinases have been profiled. This provides a unique opportunity to further improve kinase motif definitions in ELM by integrating these SPOT arrays with existing experimentally identified phosphosites.

Receptor tyrosine kinases (RTKs) regulate proliferation and actin cytoskeleton remodelling in response to extracellular signals. RTKs become autophosphorylated in tyrosine residues (pTyr) and relay the signal to non-receptor Src-family kinases (SFKs) (92) and adaptor proteins such as NCK1/2 (93), among others. This first step is achieved through binding of pTyr motifs to Src-homology 2 (SH2) domains present in SFKs and NCK1/2. The human proteome contains over 100 SH2 domains which bind defined ligands but present a significant degree of cross specificity (94,95). The present ELM update adds an extensive revision of the NCK (LIG_SH2_NCK_1) and SFK (LIG_SH2_SFK_2) specificity classes, as well as a new class describing the autoinhibitory interaction involving the C-tail of SFKs (LIG_SH2_SFK_CTail_3). The loss of the C-tail interaction leads to oncogenic activation in v-SRC. Viral and bacterial pathogens also mimic SFK and NCK SH2 motifs to induce cell proliferation and actin cytoskeleton remodelling (96).

### SLiMs in innate immunity

The human cell must be able to respond to many different types of pathogens and the tell-tale signs of infection that they provide (97). Within the cytosol of the cell, these can include both DNA (cGAS-STING) or dsRNA detectors (RIGI-MAVS) that control whether interferon regulatory factors (IRFs) can activate transcription (98,99). However, cells that are not already primed to expect pathogen invasion are most likely to succumb to the invader. Additionally, therefore, the cell surface is equipped with Tumour necrosis family receptors, Toll-like receptors, Interferon receptors and so forth so that it can be alerted to the presence of pathogens in the body and rapidly initiate defensive signalling internally, including transcriptional activation of critical defence genes such as interferons and cytokines by transcription factors such as NF-κB (97,100).

The canonical NF-κB activation pathway begins by an activated TNF superfamily receptor trimerising and recruiting a combination of TRAF family E3 ligases via TRAF-binding motifs such as PxQ variants in the cytosolic tails (Table 2). Unlike most E3 ligases, the Lysine-63 ubiquitin chains generated by the TRAFs are activating, rather than destructive signals. The pathway proceeds via IKK protein kinase activation and IκB protein kinase degradation, ultimately releasing the NF-κB RelA component for nuclear entry and gene activation. The non-canonical pathway involves TNFR recruitment of a partially distinct subset of TRAFs and ultimately releasing the NF-κB component RelB into the nucleus for gene activation (101). These pathways involve communication between several multiprotein complexes but are typically shown as rather linear.

The RIGI-MAVS detection systems for cytosolic pathogen dsRNA are present on the mitochondrial membrane's outer surface (102). The cGAS-STING detection systems for cytosolic pathogen DNA are also present on the mitochondrial outer membrane (but also the endoplasmic reticulum) (99). Both of these systems control interferon regulatory factors, using the phosphorylated LxI (pS) SLiM (Table 2) to keep the IRFs in the cytosol as long as there are no pathogenic nucleic acid signals. Most of the IRF protein family have the same SLiM and, when phosphorylated by TBK1 or IKKϵ at the SLiM itself, they homodimerise and escape the cytosol into the nucleus where they can bind and oligomerise the general transcription factors P300/CBP and together stimulate interferon gene transcription (103).

**Figure 4.**
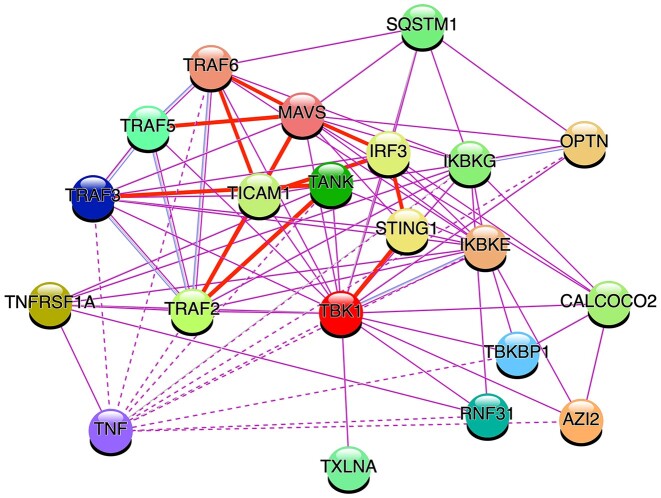
Protein interaction network centred on the key anti-pathogen and inflammatory kinase TBK1 generated by the STRING resource (122). The network shows how TBK1 plays a core role in bringing together the TRAF and IRF3 signalling systems. Edges with SLiMs annotated in ELM are shown by thick red lines. TNF (extracellular) interactions with cytosolic proteins are indirect (dotted lines). STRING settings for building the interaction network were as follows: only experimentally identified interactors of TBK1 and the 1st shell of ‘no more than 20 interactors’ in max number of interactors.

There are many complications to the innate immunity systems simplistically described above - for example, they connect to each other via some of their SLiM-mediated interactions. One of the strongest connections is through the TANK Binding Kinase (TBK1). Currently, the only biophysically identified SLiM binder to TBK1 is STING, which interacts via a TBK1 docking motif (DOC_TBK1_STING_1) (104). The acronym TANK is derived directly from NF-κB regulation (TRAF family member-associated NF-kappa-B activator), but TANK and TBK1 are reported to be core components of an IRF3 activation complex (105). Figure 4 shows a network of twenty close interactors with TBK1: Multiple interactions unite the NF-κB and IRF gene activation pathways. These closely connected anti-pathogen systems (98) illustrate why it is essential to think in terms of regulatory networks (where vertical pathways represent specific routes of information flow that can be controlled by any number of lateral interactions subject to other cell state conditions), as well as the fundamental importance of different SLiM classes in how network state is being modulated (2,106).

### Impact of AlphaFold on SLiM bioinformatics

AlphaFold has proved to be an effective predictor of IDRs (107). ELM annotators now routinely use available structure predictions from the AlphaFold database (108,109) as they aid assessing SLiM instance plausibility, taking advantage of properties such as estimated disorderliness (a low pLDDT score) (109). AlphaFold prediction results have recently been incorporated into LeishMANIAdb (110) to more precisely score the probability of putative SLiMs in *Leishmania* species proteomes, that might interact with host proteins.

In addition there is increasing interest in using AlphaFold for *de novo* modelling of putative SLiM interactions with known or potential interaction domains. For example, in a recent C degron study, AlphaFold was used to model the interaction site of a POLD2 Proline C degron on FEM1B, predicting a distinct binding site to the better studied Arginine C-degron (72). In a benchmarking study of a protein-peptide interaction dataset, AlphaFold2-Multimer achieved only 40% success rate in modelling the correct binding site and interface by default protocol. However, the accuracy was enhanced with a combination of prediction modes, which included different protocols to incorporate the peptide alignment information while modelling the protein–peptide complex (111). In a second study, an ELM-derived dataset was used to test the accuracy of AlphaFold-Multimer in modelling SLiM interactions, finding that a fragment-based approach around the interacting SLiM region can enhance the performance of modelling SLiMs (112). We expect the application of AlphaFold for SLiM prediction will be a growing area of bioinformatics research.

## Conclusions and future perspectives

Research into the biology of SLiMs continues its steady expansion. SLiMs underlie much of the complexity in cell regulation (106,113,114). Additional classes of SLiMs are being revealed while the depth of understanding accrues for the more established SLiMs. The importance of SLiMs in disease and infection continues to be better appreciated (61,115–118). Despite these advances, we consider that the current set of experimentally defined SLiMs only represent a small fraction of the ‘SLiMome’ in eukaryotic proteomes (12,36). The adoption of more expansive experimental technologies can identify novel SLiMs and feed them into more directed experimentation allowing further refinement of the recognition patterns for incompletely defined motifs. In addition, artificial intelligence approaches such as AlphaFold hold out the tantalising promise of playing a pivotal role in *de novo* computational prediction of new SLiM candidates together with their interacting protein domains (119,120). This might all mean that resource providers and funders need to work together to further enhance existing tools, develop new ones and better integrate the SLiM resources with each other and with other bioinformatic and biomedical platforms in the way that, for example, InterPro has recently connected with ELM (121). Only then can the research community fully benefit from knowledge bases such as ELM and the enormous efforts that so many have contributed to their development.

## Data Availability

ELM is available at: http://elm.eu.org.

## Notes

Present Address: Signal Transduction Laboratory, National Institute of Environmental Health Sciences, 111 TW Alexander Drive, Durham, NC 27713, USA
